# Elimination of Schistosomiasis Mekongi from Endemic Areas in Cambodia and the Lao People’s Democratic Republic: Current Status and Plans

**DOI:** 10.3390/tropicalmed4010030

**Published:** 2019-02-07

**Authors:** Virak Khieu, Somphou Sayasone, Sinuon Muth, Masashi Kirinoki, Sakhone Laymanivong, Hiroshi Ohmae, Rekol Huy, Thipphavanh Chanthapaseuth, Aya Yajima, Rattanaxay Phetsouvanh, Robert Bergquist, Peter Odermatt

**Affiliations:** 1 National Center for Parasitology, Entomology and Malaria Control, Ministry of Health, Phnom Penh 12100, Cambodia; sinuonm@gmail.com (S.M.); kolhuy@gmail.com (R.H.); 2 Lao Tropical and Public Health Institute, Vientiane 01030, Laos; somphou.sayasone@yahoo.com; 3 School of Medicine, Dokkyo Medical University, Mibu, Shimotsuga, Tochigi 321-0293, Japan; kirinoki@dokkyomed.ac.jp (M.K.); rsa40370@nifty.com (H.O.); 4 Centre for Malariology, Parasitology and Entomology, Vientiane 01000, Laos; sakhone07@gmail.com; 5 World Health Organization, Vientiane Office, Vientiane 01160, Laos; chanthapaseutht@who.int; 6 World Health Organization, Western Pacific Regional Office, Manila 1000, Philippines; yajimaa@who.int; 7 Department of Communicable Disease Control, Ministry of Health, Vientiane 01130, Laos; rattanaxay@gmail.com; 8 Department of Epidemiology and Public Health, Swiss Tropical and Public Health Institute, P.O. Box 4002 Basel, Switzerland; robert.bergquist@yahoo.se (R.B.); peter.odermatt@swisstph.ch (P.O.); 9 University of Basel, P.O. Box 4001 Basel, Switzerland

**Keywords:** *Schistosoma mekongi*, *Neotricula aperta*, snail, Cambodia, Lao PDR, elimination

## Abstract

The areas endemic for schistosomiasis in the Lao People’s Democratic Republic and in Cambodia were first reported 50 and 60 years ago, respectively. However, the causative parasite *Schistosoma mekongi* was not recognized as a separate species until 1978. The infection is distributed along a limited part of the Mekong River, regulated by the focal distribution of the intermediate snail host *Neotricula aperta*. Although more sensitive diagnostics imply a higher figure, the current use of stool examinations suggests that only about 1500 people are presently infected. This well-characterized setting should offer an exemplary potential for the elimination of the disease from its endemic areas; yet, the local topography, reservoir animals, and a dearth of safe water sources make transmission control a challenge. Control activities based on mass drug administration resulted in strong advances, and prevalence was reduced to less than 5% according to stool microscopy. Even so, transmission continues unabated, and the true number of infected people could be as much as 10 times higher than reported. On-going control activities are discussed together with plans for the future.

## 1. Historical Background

The parasitic, trematode genus *Schistosoma* puts more than 800 million people in the world’s tropical areas at risk, infecting a third of them [1,2]. Six different species of *Schistosoma* can infect humans, each depending on a specific snail species acting as intermediate host. The various endemic areas for the three main schistosome species have long been well-known, with basically *S. mansoni* in Africa and Latin America, *S. haematobium* in Africa, and *S. japonicum* in China and The Philippines (formerly also in Japan).

The adult schistosomes are miniscule worms with a preference for abdominal capillaries of the definitive human host, where they release a large number of eggs. These are excreted with either urine or feces (which route depends on the schistosome species) and infect the intermediate snail host, which releases many cercariae—a later developmental stage—into the water. The parasite’s life cycle is completed when the definitive human host comes into contact with water containing schistosome cercariae that can penetrate the human skin. However, large numbers of parasite eggs fail to be excreted and, instead, cause microscopic lesions due to the host immune reactions in various organs, most often the liver. Generally, this leads to a chronic disease with comparatively low direct mortality. Schistosomiasis as a whole constitutes one of the neglected tropical diseases (NTDs) selected for elimination by the World Health Organization (WHO). Owing to the limited geographical distribution of *Schistosoma mekongi* to endemic areas in Cambodia and the Lao People’s Democratic Republic (Lao PDR), strategies aiming at its elimination and eventual eradication can be implemented more effectively than for other, more widespread species.

Before effective chemotherapy became available in the late 1970s, the cornerstone for schistosomiasis control was broad-spectrum molluscicides directed at the intermediate snail host. However, when the drug praziquantel was introduced [3] and started to be used (at 40 mg/kg) for mass drug administration (MDA), it soon replaced most other control activities thanks to safety, high efficacy against the adult parasite worm, and easy administration [1,4]. Praziquantel changed the focus from infection prevention to morbidity reduction, reflected in a decline of the disability-adjusted life years (DALYs) metric for schistosomiasis [5,6]. This decline has, however, been contended since minor, so-called subtle morbidities are not considered by the DALY [5,6].

After schistosomiasis had been discovered in the Mekong River Basin (MRB), first in Lao PDR in 1957 [7] and 10 years later (1968) in Cambodia [8], biological research conducted in the 1970s demonstrated that the eggs from the MRB schistosomes were morphologically different from *S. japonicum* [8]. Furthermore, the former species had a different intermediate snail host [9,10] that could not infect water buffaloes [8] but was found in dogs [11]. By 1978, it became clear that the parasite was sufficiently different from *S. japonicum* to be named a separate species, *S. mekongi* [12]. Schistosomiasis mekongi is only found in specific areas along the MRB as it transverses Lao PDR and Cambodia. Due to the specific environmental variables required by its intermediate host snail, *Neotricula aperta* [9,10,13], transmission of *S. mekongi* is highly focal [14,15]. Compared to other schistosome species, the endemic areas for this kind of schistosomiasis are very limited, and the population at risk is unusually small, comprising only an estimated 150,000 people [14]. However, infection and re-infection sustain the disease, particularly in children, due to their high level of water contact [16,17]. Reservoir hosts, also play a role in maintaining the infection in the environment, although their diversity is not as broad as that seen with *S. japonicum*.

This paper reviews the work carried out after the rediscovery of the *S. mekongi* foci in the early 1990s in Lao PDR and Cambodia. We start with the historical background and continue by summarizing early control activities in each country. Subsequently, we list the achievements and elaborate on the organizational set-up, highlighting most relevant operational research activities. This review also features the more recent switch from morbidity control to elimination, followed by a discussion of the steps to achieve and the challenges involved.

### 1.1. Lao PDR

The first schistosomiasis mekongi case was diagnosed in Saint Joseph Hospital in Paris in 1957 [7], where an 18-year old Laotian patient was hospitalized, following an episode of severe hematemesis. The patient had advanced hepatosplenic pathology, and the infection was eventually traced back to his first years of life spent on Khong Island, Champasack Province, Lao PDR. Later on, a scientific paper reported on several other schistosomiasis patients originating from the same area [18]. In 1967, a WHO mission was sent to Champasack confirming the infection risk and identifying a transmission focus [19]. High village prevalence rates of schistosomiasis (up to 60%) were observed in certain districts, such as Khong and Mounlapamok in Champasack Province. Severe hepato-biliary morbidity associated with *S. mekongi* infection was frequently seen at the local health facilities. However, further follow-up studies could not be done at this point in time due to war and civil unrest in the 1970s and 1980s. In 1989, the Ministry of Health (MoH) initiated its first chemotherapy-based intervention with support from WHO in all of the endemic communities in Khong and Mounlapamok [14]. It was found that one third of all children tested were positive for *S. mekongi*, leading to the recommendation to implement health information, education, and communication (IEC) in addition to chemotherapy. This type of intervention was performed annually until 1995 and subsequently continued up to 1999 with support from the German Pharma Health Fund. After several annual MDA rounds with praziquantel, the prevalence of schistosomiasis in sentinel villages was as low as 2% [20].

### 1.2. Cambodia

In the late 1960s, schistosomiasis patients also started to be diagnosed at Phnom Penh’s Calmette Hospital in Cambodia [21,22]. All those patients originated from Kratié Province where the presence of a transmission focus was confirmed in primary surveys [23,24]. An extended survey, including the examination of 3, 767 primary school-children in villages along the Mekong River from Strung Treng, towards the Vietnamese border, discovered variable rates of infection with the highest infection (~34%) in Kratié Province, notably lower rates in Stung Treng Province (4%) and no infections in the provinces further downstream [19]. However, intradermal sensitivity tests against *S. japonicum* antigen were positive in some children (<10%) from some downstream villages, and some exposure to the parasite was documented [19].

Early observations on Khong Island in Lao PDR confirmed severe clinical manifestations of the infection [25], such as portal hypertension with dilated superficial abdominal veins or advanced ascites and/or hepatomegaly and/or splenomegaly. Adolescents and young adults were the most heavily affected. Infection rates of 60% and higher were diagnosed with co-infections with *Opisthorchis viverrini* (64%) and hookworm (44%) being very common. However, no mortality was reported [25,26]. In Cambodia, the dramatic, historical events (Cambodian Civil War) that gained momentum in the late 1960s, deterred further studies. Schistosomiasis was only brought back into the national health agenda in 1992, when the non-governmental organization (NGO) ‘Action Internationale Contre la Faim’ diagnosed marked hepato-splenomegaly in 50% of 120 schoolchildren from Ampil Tuk, a village in Kratié Province [16].

When large-scale monitoring in 1994 resulted in the diagnosis of many severe cases in 20 villages in Kratié Province, the enormity of the schistosomiasis problem in the country became fully recognized [17]. A pilot schistosomiasis control program, mainly based on MDA and IEC, was started in 1995 in Kratié Province [15]. Two years later, the program was scaled up to include all endemic districts in the two hardest hit provinces, Kratié and Stung Treng, bringing the total number of villages to 114 (56 in Kratié and 58 in Stung Treng) with an estimate of 80,000 people at risk [20]. During 1994–1995, several surveys in accessible villages along the Mekong River in Kratié Province showed infection rates between 1% and 68% [17]. Based on the initial epidemiological observations, the Cambodian MoH and the health authorities in Kratié Province, in collaboration with “Médecins sans Frontières”, initiated a rehabilitation program at Kratié provincial hospital and the district hospital of Sambo, with integrated community-based and hospital-based schistosomiasis control [15]. In 1996, Hatz et al. [20,27] conducted the first ultrasound profiling of *S. mekongi* infection in the Stung Treng Province in Cambodia, detecting pathological changes in 84% of the 299 participants with periportal thickening in 16% and parenchymal changes in 9% of those investigated.

## 2. From Morbidity Control to Elimination

### 2.1. Lao PDR

The first National Policy and Strategy for Control of Helminth Infections was developed and endorsed by the MoH in 2009 [28]. It served as a backbone for the helminth control program, targeting four groups of helminth diseases with public health significance, i.e., lymphatic filariasis (LF), soil-transmitted helminthiasis (STH), food-borne trematodiasis, and schistosomiasis.

#### 2.1.1. Policy, Commitment, and Interdisciplinarity

In 2015, the National Policy and Strategy for Control of Helminth Infections was revised and extended to cover all NTDs with public health significance in Lao PDR, such as leprosy. Following the Policy and Strategy directives, the National Committee for NTD control was established. It included members from the MoH, such as the Department of Communicable Disease Control (CDC), the Lao Tropical and Public Health Institute (Lao TPHI), the National Centre of Malariology, Parasitology and Entomology (CMPE), National Centre for Environmental Health and Water Supply as well as representatives from other administrative authorities, such as the Ministry of Education and Sports, the Ministry of Agriculture and Forestry and the Ministry of Transport and Construction. Chaired by the Deputy Minister of Health, this committee guides, monitors, advocates, and authorizes all NTD control activities in Lao PDR including the MoH budget. It also comprises all activities regarding schistosomiasis control. Its status was updated and its list of members renewed in 2018.

A specific national schistosomiasis elimination action plan for the period 2016–2020 has been developed as a guidance for the National Control Program. This plan is supported by a Technical Taskforce at the central, provincial and district levels, and the taskforce members are experts from the ministries, which are already involved by the National Committee for NTD Control.

#### 2.1.2. Activities after the Millennium Shift

**Figure 1:**
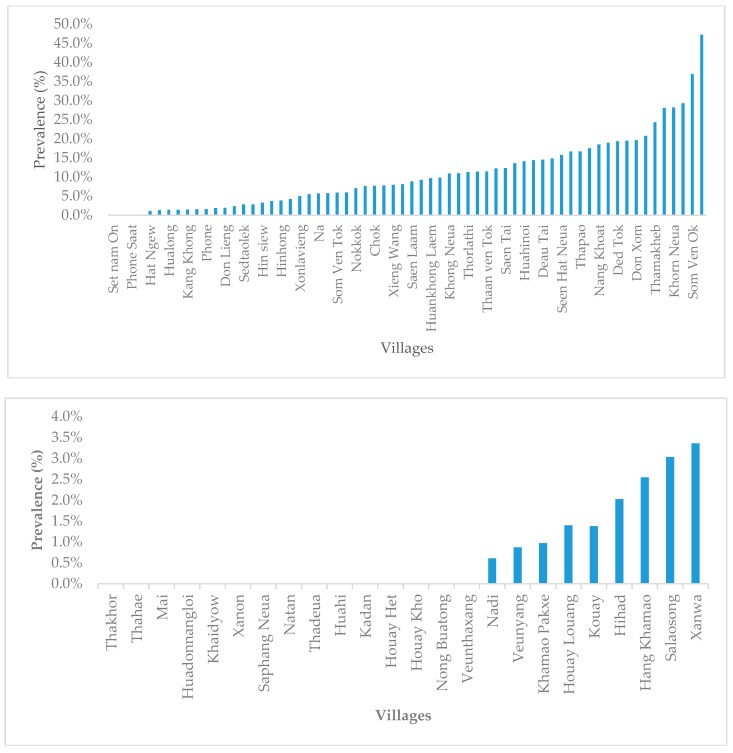
Prevalence of schistosomiasis in Khong (top) and Mounlapamok (bottom), detected by an approach based on a single Kato-Katz smear within the MoH and World Health Organization (WHO) survey in 2003.

With MDA and IEC discontinued after 1999 due to waning financial support, *S. mekongi* infection prevalence started—unsurprisingly—to rapidly increase. In 2003, a survey was conducted by the MoH, with support from WHO, using the Kato-Katz technique based on a single stool sample. It revealed an overall infection prevalence of 11.0% across the 64 endemic communities in Khong District, varying from 0% to 47.2%. The average infection prevalence in Mounlapamok District was 0.7%, only reaching higher levels (3.5%) in the most highly infected village, and with a majority of villages still completely negative (Figure 1). This re-emergence was later confirmed by a joint Lao–Swiss research project conducted by the Lao TPHI in the period 2005–2006. It showed a *S. mekongi* infection prevalence of 68% and 4% in Khong and Mounlapamok, respectively [29]. These rebounding prevalence rates in the two endemic districts brought infection rates up to the levels common before control was initiated in 1989.

**Figure 2:**
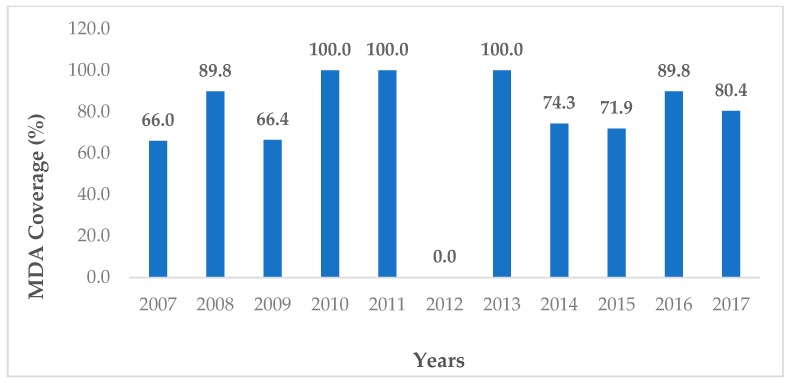
Mass drug administration (MDA) coverage at the *S. mekongi* endemic communities in Lao PDR in the period 2006–2017.

**Figure 3:**
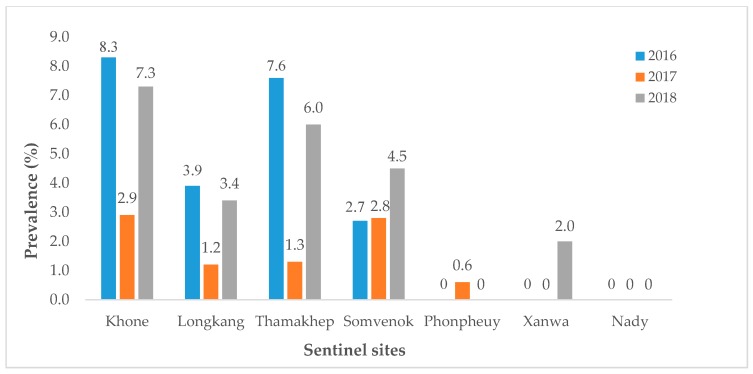
*Schistosoma mekongi* infection prevalence at the seven sentinel sites in the districts Khong and Mounlapamok in the period 2016–2018.

In 2007, the Lao MoH, in collaboration with WHO and other partners, re-established a second chemotherapy-based intervention scheme with the aim to eliminate schistosomiasis as a public health problem by bringing infection intensities below 1% in all areas, using seven specifically defined sentinel villages to monitor the intervention success (MoH, Technical Report on schistosomiasis control program, unpublished). To achieve this goal, WHO recommended maintaining the annual MDA with praziquantel covering at least 75% of each community treated. The high-risk population, namely school-aged children and adults, i.e., people of ages from 5 to 60 years old, were targeted. They conducted ten rounds of MDA, with an average coverage of >80%, which brought the prevalence down to less than 10% in 2016 at all the sentinel sites, with no cases of high-intensity infection (>400 eggs per gram) detected (Figure 2) [30]. In 2017, the infection prevalence was less than 3%, with only 0.1% of them being high-intensity infections. In 2018, less than 6% of villagers at most sentinel sites were infected, with an overall prevalence of 3.2%. No patient with high-intensity infection was diagnosed (Figure 3). In addition, spot-checks were conducted in 20 randomly selected villages with a total of 3, 533 study participants. In 2017, and thus far in 2018, the average infection prevalence reached only 0.7%. No high-intensity infection was diagnosed [31], but continued MDA in the future is planned.

With regard to investigating if transmission involving reservoir hosts could become a problem for elimination of schistosomiasis mekongi, Strandgaard et al. [32] conducted a survey focused on domestic pigs in the Khong District. Working with a total number of 98 pigs, detection of *S. mekongi* eggs in the liver, intestines, and stools of 12 (12.2%) of them confirmed this animal as a possible definitive host.

### 2.2. Cambodia

**Figure 4:**
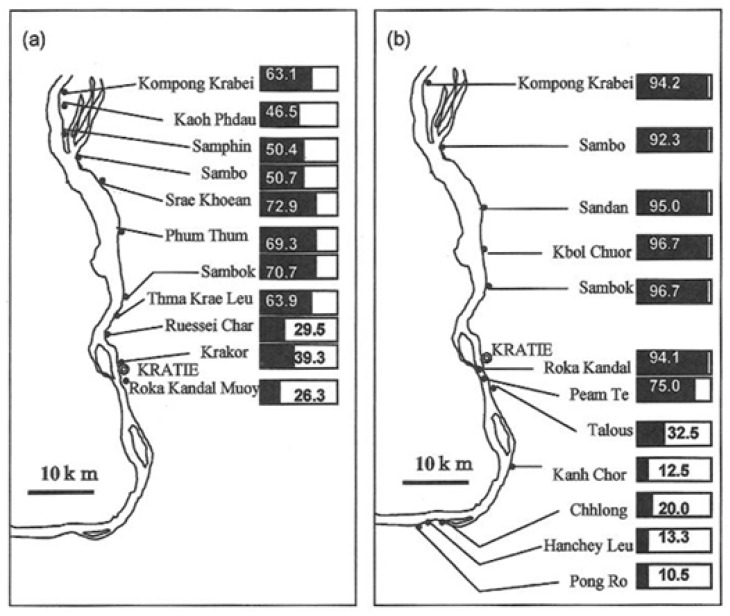
Comparison of results of stool examination and ELISA conducted in villages along the Mekong River in the Kratié Province [14]. Positive ratios (%) are represented by the numbers in the bar charts. (**a**) Prevalence of schistosomiasis mekongi, as determined by stool examination during 1994–1995 [17], and (**b**) prevalence of schistosomiasis mekongi, as determined by ELISA using *S. japonicum* soluble egg antigen (SEA) in the period 1997–1998 [14].

In collaboration with the MoH National Center for Parasitology, Entomology and Malaria Control (CNM) in Phnom Penh, researchers from Dokkyo Medical University, Mibu, Tochigi, Japan have been conducting epidemiological surveys in Cambodia since 1997. The aim was to elucidate the status of schistosomiasis due to *S. mekongi* [14]. Figure 4 shows the results of a seroepidemiological survey conducted in 1997 and 1998, using the enzyme-linked immunosorbent assay (ELISA) with *S. japonicum* soluble egg antigen (SEA) according to Matsuda et al. [33]. The results were consistent with the stool examinations with regard to the distribution of the infection among the endemic villages [14]. However, egg-positive rates exceeding 50% and ELISA-positive rates higher than 90% were recorded in some villages in the northern part of Kratié Province, while ELISA-positive rates of less than 30% were recorded in some villages in the southern part of the area [14]. None of these parts of Kratié Province had been targeted by surveillance prior to 1997; consequently, no stool examinations had been performed there in the period 1994–1995.

Serology surveys were part of the diagnostic approach, owing to their usefulness for the detection of schistosomiasis risk by village, particularly in low-endemic foci. Since 2003, ELISAs specific for *S. mekongi* were applied, relying on a technique using sodium metaperiodate (SMP) to reduce non-specific cross-reactions via oxidization of polysaccharide residues in the antigen molecules [34]. Ultrasound, a technique showing severe pathological changes in the liver that are often irreversible, was carried out using portable devices. However, this kind of examination can only be used for personal examination as well as for regional risk monitoring and historical evaluation [35]. Such examinations were conducted in 2003 to compare morbidity due to *S. mekongi* infection in villages in Kratié Province characterized by high and low endemicity [36].

**Figure 5:**
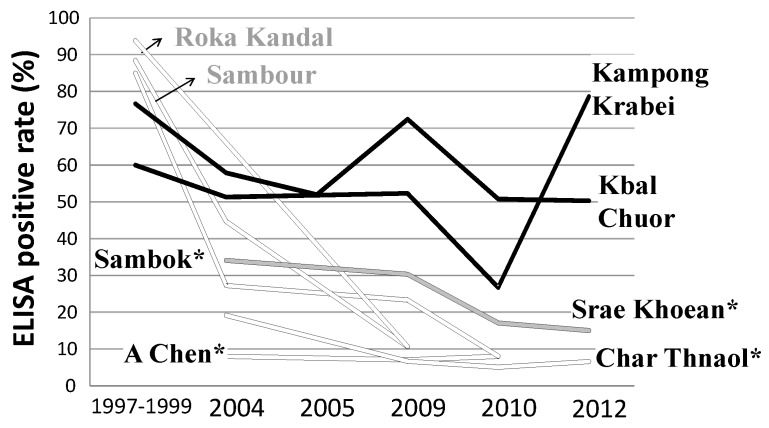
Changes in specific antibody rates in villages in the Kratié Province, Cambodia, during the period 1997–2012. Legend: *Sentinel villages for monitoring, as designated by the National Center for Parasitology, Entomology and Malaria Control (CNM). Black line: high-risk villages (≥50%); grey line: Moderate-risk villages (≥10% and <50%); white line: low-risk village (<10%) SMP–ELISA using *S. mekongi* SEA.

Stool examinations were carried out using the Kato-Katz technique [37]. The SMP–ELISA technique was used for surveys in sentinel villages designated by the National Schistosomiasis Control Program (in Achen, Char Thnaol, Srae Kheun and Sambok in Kratié Province as well as several additional spot-check sites). As shown in Figure 5, ELISA-positive rates in sentinel villages and at two additional sites (Roka Kandal and Sambour) dramatically decreased to below 20%, while ELISA-positive rates remained high (>50%) at two spot-check sites (Kampong Krabei and Kbal Chuor).

Applying ultrasound examination, dilatation of the portal vein was detected in 139 of 366 participants (38%) in a high-endemic village group and in 10 of the 117 participants (1.2%) in low-endemic villages. The characteristic ultrasound pattern of septum formation in the liver parenchyma producing the fish scale pattern noted in *S. japonicum* infection, was not observed in *S. mekongi* infections. However, in the 1990s, splenomegaly due to *S. mekongi* infection was reported to be more severe than that due to *S. japonicum* infection [38,39].

Various animals have been suspected to act as reservoir infection sources. While pigs have been experimentally shown to be possible natural reservoirs of *S. mekongi* in Lao PDR [32], this finding could only be confirmed for dogs in Cambodia. Natural schistosome infection in dogs in Cambodia was first reported by Matsumoto et al. in 2002 [40]. Schistosome eggs were detected in 1 of the 28 canine stool samples (3.6%) collected from Kbal Chuor village in Kratié Province in 2000 [40]. During a more recent survey in 2010, 15 and 17 canine stool samples were collected from two villages, Kbal Chuor and Kampong Krabei, in Kratié Province; *S. mekongi* eggs were detected in 2 (13.3%) and 1 (5.9%) of the samples from these villages, respectively.

Hisakane et al. [41] constructed a mathematical model for *S. mekongi* transmission in Cambodia, according to which dogs were considered definitive hosts in addition to humans. The simulations indicated that biannual universal and/or targeted treatment could reduce the prevalence to below 5%, within 8 years, based on 85% coverage of the residents [41]. Natural *S. mekongi* infections were not detected in cats, pigs, cows, water buffalos, horses or rats in Cambodia [40]. Rodents have been shown to be susceptible to *S. mekongi* by experimental infection; however, no natural infections have been detected to date. In 2016, ten *S. mekongi* infected *N. aperta* were found in 4840 corrected mollusks (0.2%).

A national task force for the control of STH, schistosomiasis and LF was set up in 2003. The members of the committee were representatives of different departments, ministries, and NGOs. Each department, institution and ministry involved has the responsibility to contribute to specific control/elimination activities. While CNM is responsible for developing the control/elimination strategies of NTDs, including *S. mekongi*, the Department of School Health of the Ministry of Education, Youth and Sports manages health education and support of water, sanitation and hygiene (WASH) approach in schools, the Department of Rural Health of the Ministry of Rural Development (MRD) is in charge of WASH in the communities, and WHO and the United Nations Children’s Fund (UNICEF) offer technical and financial support. In 2004, the first National Policy and Guidelines for Helminth Control in Cambodia was established and adopted by the National Task Force for the Control of STH, schistosomiasis and LF [39].

## 3. Achievements

### 3.1. Lao PDR

The move from control to elimination is a challenge and recent experience suggests that interruption of MDA without adequate sanitary improvements, would within a few years lead to the parasite reclaiming its previous high endemicity. With this in mind, the MoH has started community-led initiatives to eliminate schistosomiasis by combining MDA with schistosomiasis-adapted Water, Sanitation and Hygiene (WASH) interventions (CL-SWASH) in two pilot villages in 2015, with technical support from WHO and other partners [31,42,43,44]. CL-SWASH integrates various ongoing MoH components of parasitic infection control with the aim to expand development of Water Safety Plans –a multi-risk management approach ranging from community participation to nationwide activities. Communities will be empowered to self-assess their environmental health risk situation, particularly in relation to schistosomiasis transmission, for instance, by interrupting transmission by eliminating open defecation. CL-SWASH continues to expand and had completed activities in 24 villages by the end of 2018, as well as outlined a plan to cover all 202 endemic villages by 2025. In addition, the MoH national action plan for elimination of schistosomiasis reflects the recommendation by the ‘Expert Consultation to Accelerate Elimination of Asian Schistosomiasis’ conducted in Shanghai, China under the auspices of the WHO Regional Office for the Western Pacific (WPRO) in May 2017 [45]. The main conclusion was to shift activities from the current chemotherapy-only intervention to an integrated One-Health strategy, aiming to interrupt transmission by 2025 and to achieve certified elimination by 2030 [46].

### 3.2. Cambodia

**Figure 6:**
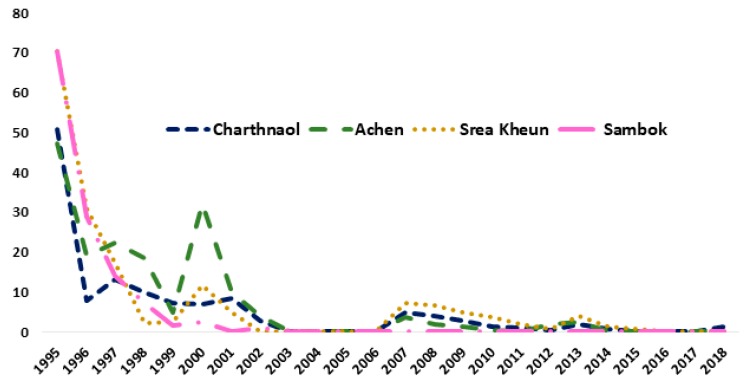
*S. mekongi* prevalence distribution in four sentinel site villages of Kratié, Cambodia 1995–2018.

Since 1995, the MDA together with the IEC campaigns have been conducted annually in the two provinces endemic for *S. mekongi*. In Kratié Province, four sentinel villages were followed-up annually. Figure 6 shows the rapid decrease in the prevalence of *S. mekongi* infection from 1995 to 2018. The *S. mekongi* prevalence at four sentinel surveillance sites in Kratié Province dropped dramatically from 70% in 1995 to less than 1% in 2018. According to the CNM annual reports no new patients with *S. mekongi* infection were diagnosed in these villages over the last few years, demonstrating the positive impact of the intervention.

In 2016, an external evaluation of schistosomiasis control in Cambodia was led by WHO and based on the formalin-detergent diagnostic method [47] proven to have higher sensitivity than the standard Kato-Katz method [37]. The evaluation demonstrated absence of high-intensity infections, both at the sentinel sites and the two additional spot-check sites [48]. Based on this finding, it was concluded that annual rounds of MDA, targeting the entire at-risk population above 5 years of age would be sufficient to achieve elimination of schistosomiasis as a public health problem defined as <1% prevalence of high-intensity infection. This success encouraged the MoH to shift gear from disease control to elimination in alignment with the recommendation of the 2017 WHO Expert Consultation [45]. In 2018, the National Strategic Plan for Elimination of Schistosomiasis (2019–2023) was developed after holding a national consultation workshop with all relevant stakeholders at the national and provincial levels, with input from MoH, MRD, Ministry of Agriculture, Forestry and Fishery, and the Ministry of Education, Youth and Sports. The aim is to interrupt transmission of schistosomiasis by 2025 and validate elimination of schistosomiasis by 2030. Three main elimination strategies were adopted:

1. Universal access to the One-Health intervention package consisting of preventive chemotherapy, CL-SWASH, and treatment of the animal reservoirs;
2. Strengthening community members’ health literacy to prevent reinfection and interrupt transmission through a sustained change of sanitation and hygiene behaviour empowering people to act as drivers of schistosomiasis elimination; and
3. Adoption of effective and sustained active and passive surveillance of schistosomiasis in human and reservoirs hosts.

## 4. Next Steps and Challenges

The prevalence information in Lao PDR and Cambodia discussed here, is based on stool examination by the Kato-Katz technique [49], often based on a single stool sample, which implies that the real prevalence could be considerably higher than that presented. While the use of the Kato-Katz technique is acceptable in areas characterized by high-intensity of infection, the recent reduction of *S. mekongi* intensity of infection, following regular MDA with praziquantel, requires a rapid switch to more sensitive diagnostic techniques [49].

The polymerase chain reaction (PCR) and the loop-mediated isothermal amplification (LAMP) are highly sensitive and specific diagnostic assays that have been validated for schistosomiasis diagnosis [50,51]. The LAMP technique holds the advantage of being applicable in field laboratories, where it has been used to detect *Schistosoma* in the snail host [52,53], an application that should be useful for monitoring transmission. Detecting circulating schistosome antigens (cathodic circulating antigen, CCA or anodic circulating antigen, CAA) in sera from infected humans [54] represents a different approach. An added benefit is that these antigens pass from the blood circulation into the urine, allowing the testing of urine samples rather than blood [54], which should make people more receptive to the recurrent testing that will be needed in the future. A commercial point-of-care (POC-CCA) test for *S. mansoni* has delivered excellent results in Africa [55,56], identifying three to four times more infected individuals compared to the Kato-Katz technique [56]. When POC-CCA and a CAA test were compared with the Kato-Katz stool examination in Lao PDR and Cambodia, the two former assays showed 3- and 6-times better sensitivity, respectively [57]. However, cross-reactivity with other intestinal trematode infections, such as *O. viverrini*, cannot be ruled out, necessitating extended evaluations. Thus, before these tests can become standard assays in control programs, more experience with them are needed. Efforts in this direction are on-going.

The national helminth control programs in Cambodia and Lao PDR implement schistosomiasis control activities independently; however, the two teams regularly visit each other to gain insights into the operational activities and implementation of each program. This is important for sustaining the goals set, and needs now to be complemented by a database encompassing the entire area endemic for *S. mekongi*, distributed to these two countries. The preliminary database, established in Lao PDR to keep track of ongoing control activities and impact measures implemented, has proven useful by contributing to the adoption of standardized measures of infection and morbidity. An online database accessible by all stakeholders would be instrumental for exchange of surveillance data and rapid response action when needed, substantially facilitating the work towards elimination of *S. mekongi* infections. Once a *S. mekongi* database has been established, the information can be leveraged by bundling the data together with cartographic records and remotely sensed data from earth-observing satellites, displaying the information in a geographical information system (GIS) [58,59]. Thanks to the growing accessibility to the Internet and global positioning systems, relevant data can be collected from satellite sensors and analyzed in field settings, or other resource-poor environments, by laptop computers, and even mobile phones.

The multi-sectoral control approach, initiated in pilot villages in both countries, has thus far demonstrated both feasibility and suitability. Nonetheless, scaling up this kind of intervention is a challenge that will require substantial efforts when enlarging activities to cover communities and higher levels. Each endemic village initially requires six provincial-level officials from the different administrative sectors, involved to spend three working days initiating the activities needed. Later all enrolled villages receive follow-up visits to consolidate the various activities started. However, the resources needed, particularly with reference to trained personnel, to implement multi-sectoral activities in all villages endemic for schistosomiasis (114 in Cambodia and 202 in Lao PDR) are currently not available. This notwithstanding, the local health services must become more adapted to surveillance-and-response systems [60]. Today, these services do not have a defined role, neither with respect to diagnosis nor to treatment delivery. Strengthening of the health system has been recognized to be essential for the long-term success of control. Consequently, defining the role of curative and preventive health services in the surveillance and response, and diagnoses and treatment of *S. mekongi* and improving these services accordingly will be indispensable for successful control and future elimination.

In the absence of a schistosomiasis vaccine or alternative drugs, praziquantel has now been used as the mainstay for control, wherever possible complemented by WASH or CL-SWASH. Today, praziquantel treatment through annual MDA is assured as the MoHs in Cambodia and Lao PDR both make substantial efforts to maintain the annual treatment rounds. However, the move towards elimination planned will require transmission control, something which is more difficult in areas endemic for *S. mekongi* than anywhere else. Even if sufficiently sensitive snail diagnostics exist, the collection of specimens for testing is a challenge as the average shell length of *N. aperta* is less than 3 mm [60]. Further, the snails are restricted to shallow areas with water moving fast over wood or stone surfaces. As such conditions only exist during the dry season, the snails mostly originate from eggs laid the previous year [61]. Thus, every year provides new snail populations and they can only be found during a few months in the first half of the year. Transmission control should also involve reservoir host such as dogs and other domestic animals found susceptible for *S. mekongi* infection. The most expedient way of checking these would be serology, even if antibody titers will be less significant than direct tests of circulating or excreted schistosome antigens.

## 5. Conclusions

Despite the geographically fragmented environment along the Mekong River in Cambodia and Lao PDR, it should be feasible to achieve elimination of *S. mekongi* owing to the confinement of this parasite to extremely restricted areas. In fact, *S. mekongi* has the smallest distribution of any of the schistosome species. However, elimination has proved more difficult than initially thought. Despite strong progress in a large part of the endemic area, thanks to the MDA with praziquantel and the initiation of the multi-sectoral control approach, elimination remains a distant goal. What is needed for improved control are (i) scaling-up of the multi-sectoral control approach; (ii) application of a common database; and (iii), in the longer perspective, local health services to a surveillance-and-response system. A more thorough study of which animals can act as definitive hosts, would also be useful.

These moves, however, will only be bear fruit if a reliable representation of *S. mekongi* prevalence and intensity of infection can be ensured, something that is clearly attainable through the implementation of more sensitive diagnostics, supported by remotely sensed data and GIS technology.
